# Factors influencing emergency department utilisation using Andersen’s behavioural model: a cross-sectional study in a public hospital in Malaysia

**DOI:** 10.3389/fpubh.2026.1731516

**Published:** 2026-02-12

**Authors:** Norbaidurah Ithnain, Saiful Adli Suhaimi, Evi Diana Omar, Hasnah Mat, Ahmad Tajuddin Mohamad Nor, Manimaran Krishnan

**Affiliations:** 1 Institute for Health Behavioural Research, National Institutes of Health, Ministry of Health Malaysia, Shah Alam, Selangor, Malaysia; 2 Sector for Biostatistics and Data Repository, National Institutes of Health, Ministry of Health Malaysia, Shah Alam, Selangor, Malaysia; 3 Hospital Tengku Ampuan Rahimah Klang, Ministry of Health Malaysia, Klang, Selangor, Malaysia

**Keywords:** Andersen’s behavioural model, behavioural insights, emergency department, factors, healthcare utilisation, Malaysia

## Abstract

**Introduction:**

Overcrowding in emergency departments (EDs) remains a critical issue, commonly driven by non-urgent visits associated with behavioural, socioeconomic, and system-level factors.

**Methods:**

This cross-sectional study was conducted in the emergency department of Hospital Tengku Ampuan Rahimah Klang, Malaysia, in 2023, involving convenience-sampled adult Malaysian visitors in the green zone. Respondents completed a structured questionnaire based on Andersen’s behavioural model to assess predisposing, enabling, and need factors, as well as ED utilisation patterns. Data were analysed with SPSS 29.0 using descriptive statistics and multiple logistic regression, with significance set at *p* < 0.05.

**Results:**

Among the 381 respondents, most were young adults, low-income earners, and perceived their conditions as urgent despite presenting with non-critical symptoms. In exploring the reasons for ED attendance, respondents frequently cited trust in care quality and the proximity of the facility as key reasons for choosing ED over other care options. Multiple analysis revealed that marital status, insurance coverage, and illness duration had a significant impact on attempts to access alternative services. Being married was associated with increased ED visit frequency in the past 12 months, while insurance coverage and illness duration were notably linked with ED visits. These findings highlight how predisposing (marital status), enabling (insurance coverage), and need (illness duration) factors, as framed by Andersen’s behavioural model, shape ED utilisation patterns.

**Conclusion:**

Understanding these dynamics provides a foundation for developing strategies that combine behavioural insights with stronger primary care access, patient education, and service planning. Such measures are essential to reduce non-urgent ED visits, alleviate overcrowding, and enhance the efficiency of Malaysia’s public healthcare system.

## Background

Emergency department (ED) overuse is a major global healthcare challenge, leading to overcrowding, increased wait times, and strain on limited resources. This issue is primarily driven by rising demand, notably from individuals with non-urgent conditions like mild headaches, minor cuts, and fevers, who could typically be managed at primary healthcare facilities (1–3). Factors contributing to these inappropriate visits include socioeconomic vulnerabilities such as lack of supplementary health insurance, limited awareness of alternative services like primary care, telemedicine, and private facilities, and challenges in accessing these alternatives, leading to repeated ED utilisation (4, 5). Furthermore, system-level issues such as limited hospital resources, insufficient staffing, and inefficient processes that extend patient stays exacerbate ED overcrowding (2, 6, 7).

Similar trends of non-urgent ED utilisation are observed across Southeast Asia and other low- and middle-income countries (LMICs), highlighting the universal nature of this challenge. For instance, studies in Jakarta, Indonesia, and Thailand have shown a preference for ED services even for non-critical issues, often due to limited awareness of prehospital options, lack of after-hours alternatives, convenience, and patients’ perceptions of the severity of their conditions (8–10). Within Malaysia, healthcare providers face similar significant challenges, including ED overcrowding (11, 12). Locally conducted studies have determined that misconceptions about ED usage, perceived illness severity, inability to access outpatient care during office hours, and the convenience of ED proximity are common reasons driving inappropriate ED visits despite the availability of primary care options (3, 13).

The consequences of this overcrowding phenomenon are significant, including delayed treatments, worsened patient outcomes, decreased satisfaction (7, 14), and financial strain on hospitals due to inefficiencies such as prolonged waiting periods (15). Consequently, strategies such as improving resource allocation, optimising staffing levels, and managing patient flow through predictive models are being considered (16). However, effectively addressing ED overuse requires understanding the behavioural factors that influence healthcare-seeking decisions, which goes beyond purely operational strategies.

A behavioural insights approach, which applies psychological, social, and cognitive principles, offers valuable perspectives on public perception and decision-making (17, 18). This technique can identify biases and heuristics influencing behaviour, thereby informing the design of more effective policies and targeted interventions, such as “nudging,” to improve efficiency and public engagement (17, 19). Determining the specific drivers that prompt individuals to choose the ED over alternative services is crucial for optimising healthcare utilisation, reducing unnecessary visits, and enhancing patient outcomes.

Therefore, this study integrates Andersen’s behavioural model of healthcare usage to establish the factors contributing to ED visits. This model categorises determinants into predisposing (e.g., age, gender, education), enabling (e.g., income, insurance, proximity to facilities), and need factors (e.g., perceived or evaluated illness severity) (20). By using this framework, the study aims to identify the reasons for seeking treatment in the ED, the conditions associated with attempts to use alternative services or first-time visits, and the parameters contributing to refraining from alternatives or frequent visits. The insights derived are intended to inform behaviour-based interventions and guide the development of targeted, evidence-based strategies to promote appropriate healthcare-seeking behaviours and optimise emergency service use.

## Methodology

### Study design and setting

The ED of Hospital Tengku Ampuan Rahimah Klang recorded approximately 53,360 visits in 2022, with 35.2% from the green zone. Malaysian ED services are essential for acute medical care, providing immediate intervention through a multi-tiered triage system. The triage system is based on the Malaysian Triage Category (MTC), which classifies patients into three zones: red (immediate attention), yellow (urgent but not life-threatening), and green (non-critical issues). Patients in the green zone typically have minor injuries or less severe illnesses and may experience longer waiting times as their conditions are considered stable.

In this study, a cross-sectional survey was conducted in 2023 amongst Malaysians aged 18 years and older in the green zone of the ED, including patients, their caregivers, or employers. Foreign nationals and patients admitted to the Patient Admission Centre (PAC) were excluded. Respondents were recruited at different times of the day, including weekends, using a convenience sampling method. After giving voluntary consent, participants completed a self-administered questionnaire taking approximately 5–15 min. No follow-ups were conducted.

### Data collection tool

A structured questionnaire was specifically developed for this study and informed by Andersen’s Behavioural Model of Healthcare Utilisation, which conceptualises healthcare use through predisposing, enabling, and need factors. Item development was guided by constructs from the original model and adapted from instruments used in previous emergency care and healthcare utilisation studies (3, 21) to ensure contextual relevance to emergency department (ED) settings. The final questionnaire comprised 29 items across four sections.

- Predisposing factors included age, gender, race, marital status, employment status, and educational level.
- Enabling factors captured household income, insurance status, mode of transportation to the ED, and distance from residence to the ED.
- Need factors assessed duration of illness onset and perceived urgency of treatment.
- ED utilisation patterns were measured using two dichotomous items: *“Did you seek any other alternative services before this ED visit?”* and *“Have you visited an ED in the last 12 months?.”* Types of health concerns prompting the ED visit were captured using a multiple-response item listing commonly reported acute and chronic conditions (e.g., fever, cough, injuries, chronic illnesses).

Most variables employed nominal or dichotomous response options, whilst continuous variables such as distance to the ED were recorded numerically.

### Content validity

Content validity was assessed through expert panel review involving three public emergency physicians, two public health physicians, and two medical officers with experience in ED service delivery. Experts independently evaluated each item for relevance, clarity, and comprehensiveness in relation to Andersen’s model and the study objectives. Feedback focused on item wording, redundancy, and appropriateness for the ED context. Minor revisions were made, including rephrasing ambiguous items and refining response categories, before finalisation of the instrument.

### Reliability

Reliability testing was conducted through a pilot study involving 30 participants who were not included in the main study sample. Internal consistency was assessed for multi-item measures related to reasons for ED visitation, yielding acceptable reliability (Cronbach’s *α* = 0.82). For single-item and categorical variables, test–retest reliability was evaluated over a two-week interval, demonstrating high stability with agreement exceeding 85% across items.

### Ethics and consent

This study acquired approval from the Medical Research and Ethics Committee (MREC), Ministry of Health Malaysia [NMRR ID-23-00535-JYG (IIR)]. The respondents were also informed that their participation would be voluntary and anonymous, and that it would not impact the health treatments they received. Furthermore, the respondents were provided with a consent form outlining the research aim and their rights.

### Data analysis

Data analysis in this study was conducted with SPSS 29.0. In this study, descriptive statistics were used to represent the participants’ characteristics and reasons for ED visits. Meanwhile, multiple logistic regression analysis was employed to assess factors related to ED utilisation patterns. Variables included in the multiple logistic regression model were selected based on *p*-values <0.25 from univariable analysis and guided by expert knowledge. This variable selection approach also serves as a strategy to mitigate overfitting by reducing the number of predictors in the model. The enter method was used for the multiple logistic regression model. Model calibration was evaluated using the Hosmer-Lemeshow test, with a *p*-value >0.05 indicating adequate model fit. Multicollinearity amongst predictor variables was assessed using variance inflation factors (VIF), with a cutoff value of <10, and no significant multicollinearity was detected (22). All results were considered statistically significant at *p*-value < 0.05.

## Results

Based on Table 1, a total of 381 respondents participated in this study. Most respondents were between 18 and 34 years old (*n* = 210, 55.1%). Regarding gender distribution, the number of male respondents was slightly higher (*n* = 200, 52.5%) compared to females (*n* = 181, 47.5%). Most respondents were Malays (*n* = 270, 70.9%), followed by Indians (*n* = 78, 20.5%), Chinese (*n* = 21, 5.5%), and others (*n* = 12, 3.1%). A total of 267 respondents were married (*n* = 267, 70.1%), whilst more than half worked in the private sector (*n* = 205, 53.8%). In terms of education, a considerable number had obtained secondary education (*n* = 221, 58.0%). Regarding socio-economic status, the lower income group comprised the largest proportion (*n* = 334, 87.7%), whilst only a minimal number were categorised as high-income (*n* = 1, 0.3%).

**Table 1:** The characteristics of the respondents.

Characteristic		*n* (%)
Age (years old)	18–34	210 (55.1)
	35–49	125 (32.8)
	50–64	39 (10.2)
	65 and above	7 (1.8)
Gender	Male	200 (52.5)
	Female	181 (47.5)
Race	Malay	270 (70.9)
	Indian	78 (20.5)
	Chinese	21 (5.5)
	Others	12 (3.1)
Marital status	Single	104 (27.3)
	Married	267 (70.1)
	Widower/Widow/Widowed	10 (2.6)
Employment status	Private worker	205 (53.8)
	Housewife	50 (13.1)
	Self-employed	48 (12.6)
	Public servant	44 (11.5)
	Student	17 (4.5)
	Unemployed	10 (2.6)
	Pensioner	7 (1.8)
Educational level	Tertiary (College/University)	134 (35.2)
	Secondary school	221 (58.0)
	Primary school	19 (5.0)
	No formal education	7 (1.8)
Income (RM: Ringgit Malaysia) (Classification by Department of Statistic Malaysia)	Under RM 4,850 (Low income)	334 (87.7)
	RM 4,851–RM 10,970 (Middle income)	46 (12.1)
	Over RM 10,970 (High Income)	1 (0.3)
Insurance status	Yes	132 (34.6)
	No	249 (65.4)
Transportation to the ED	Own transport	339 (89.0)
	Public transport	13 (3.4)
	Others	29 (7.6)
Distance to the ED (km)^a^		12.1 (8.1)
Time of visit	Session 1: 8.00 a.m.–2.00 p.m.	34 (8.9)
	Session 2: 2.00 p.m.–07.00 p.m.	160 (42)
	Session 3: 7.00 p.m.–12.00 a.m.	141(37)
	Session 4: 12.00 a.m.–8.00 a.m.	46 (12.1)
Duration of illness onset	Less than 24 h	192 (50.3)
	1–3 days	128 (33.6)
	4–7 days	37 (9.7)
	1–2 weeks	13 (3.4)
	3–4 weeks	11 (2.9)
Perceived need for urgent treatment	Yes	312 (81.9)
	No	69 (18.1)
Type of health concern: [Multiple responses were allowed; some respondents did not report a health concern, so total frequency may not equal the number of participants (*n* = 381)].	Fever, cough, and cold	128 (33.6)
	Accidents	38 (10)
	Injuries	23 (6.0)
	Stomach pain	17 (4.5)
	High blood pressure, diabetes, and heart conditions	13 (3.4)
	Body aches	12 (3.1)
	Leg pain	12 (3.1)
	Shortness of breath	10 (2.6)
	Dengue fever	9 (2.4)
	Chest pain	9 (2.4)
	Asthma	8 (2.1)
	Stroke	7 (1.8)
	Gastric issues	6 (1.6)
	Headaches	6 (1.6)
	Swelling of body parts	5 (1.3)
	Heart issues	5 (1.3)
	Earaches	5 (1.3)
	Diarrhoea	4 (1.0)
	Women health concerns	4 (1.0)
	Knee pain	4 (1.0)
	Allergic reactions	3 (0.8)
	Animal bites	3 (0.8)
	Jaundice	3 (0.8)
	Lung issues	3 (0.8)
	Eye concerns	3 (0.8)
	Boils	2 (0.5)
	Wound cleaning	2 (0.5)
	Loss of appetite	2 (0.5)
	Food poisoning	2 (0.5)
	Mental health issues	2 (0.5)
	Vomiting	2 (0.5)
	Suicide attempts (overdose due to depression/anxiety concerns)	1 (0.3)
	Others	23 (6.0)
Attempting an alternative service before visiting the ED	Yes	179 (47.0)
	No	202 (53.0)
The ED visit pattern within 12 months	First-time users	87 (22.8)
	Multiple-time users	294 (77.2)

^a^Represents values expressed as mean (SD).

A majority of the respondents in this study utilised their own transport to access the ED (*n* = 339, 89.0%), a minority of them employed public transportation services (*n* = 13, 3.4%), whereas a moderate percentage utilised other mode of transportation (*n* = 29, 7.6%). The mean distance of the respondents to the ED was 12.1 ± 8.1 km. Distinct patterns of patient arrival times at the ED were also observed, with the most prevalent time of total admissions recorded being from 2:00 p.m. to 7:00 p.m. (*n* = 160, 42.0%). Furthermore, a considerable proportion of the respondents sought medical care between 7:00 p.m. and 12:00 a.m. (*n* = 141, 37.0%).

Over half of the respondents in this study stated seeking medical attention within the first 24 h of experiencing symptoms (*n* = 192, 50.3%), whilst 128 patients sought medical assistance within 1–3 days after the onset of symptoms (33.6%). Conversely, only 11 respondents (2.9%), the least number, visited the ED between three and 4 weeks of experiencing symptoms. The respondents’ perspective on their medical conditions in this study was quite striking, with an overwhelming majority (*n* = 312, 81.9%) believing they required immediate medical assistance. Furthermore, fever, cough, and cold were amongst the most frequently reported health conditions (*n* = 128, 33.6%) by the respondents.

### Reasons for ED visitation

According to the data in Table 2, amongst the critical drivers for seeking treatment at a public hospital ED, the most frequently reported reasons included 24-h ED operation (*n* = 360, 94.5%), affordable treatment costs (*n* = 349, 91.6%), and confidence in the quality of care provided (*n* = 332, 87.1%). Other commonly cited reasons were accurate health assessment (*n* = 322, 84.5%), severe or urgent conditions requiring immediate care (*n* = 312, 81.9%), and satisfaction with healthcare services at the ED (*n* = 288, 75.6%). Many respondents also indicated that not needing a referral or appointment (*n* = 287, 75.3%), ED proximity to residence (*n* = 267, 70.1%), and uncertainty about managing health (*n* = 262, 68.8%) influenced their decision to seek care at the ED.

**Table 2:** The reasons for seeking treatment in a public hospital ED.

No.	Statement	Yes, *n* (%)	No, *n* (%)
1.	Seeking treatment at the ED due to severe conditions and requiring immediate care	312 (81.9)	69 (18.1)
2.	Seeking treatment at the ED because had not recovered despite receiving treatment	245 (64.3)	136 (35.7)
3.	Seeking treatment at the ED to assess health accurately	322 (84.5)	59 (15.5)
4.	Seeking treatment at the ED due to uncertainty about managing health	262 (68.8)	119 (31.2)
5.	The ED is close to residence	267 (70.1)	114 (29.9)
6.	Confident in the quality of care provided at the ED	332 (87.1)	49 (12.9)
7.	Treatment costs at the ED were affordable	349 (91.6)	32 (8.4)
8.	The ED operates 24 h	360 (94.5)	21 (5.5)
9.	Do not necessitate a referral or appointment to see a doctor at the ED	287 (75.3)	94 (24.7)
10.	Seeking treatment at the ED was typical for individuals and families with any type of illness	201 (52.8)	180 (47.2)
11.	Seeking treatment at the ED based on the advice of family members or friends	214 (56.2)	167 (43.8)
12.	Considerably satisfied with the healthcare services provided by the staff at the ED	288 (75.6)	93 (24.4)
13.	Have family members or friends working at the ED	47 (12.3)	334 (87.7)

A moderate proportion of respondents reported visiting the ED because they had not recovered despite prior treatment (*n* = 245, 64.3%), based on advice from family or friends (*n* = 214, 56.2%), or as a typical practise for themselves and their families (*n* = 201, 52.8%). Few respondents reported having family members or friends working at the ED (*n* = 47, 12.3%), indicating that personal connections were less influential in their decision.

### Factors associated with attempting alternative service before ED presentation and first-time ED visit in the past 12 months

The associations between predisposing, enabling, and need factors, and attempting alternative services before visiting the ED are summarised in Table 3. The *p*-values in the table indicate the significance of the associations. Based on the data, marital status was a notable contributor to the respondents’ visits to the ED. A lower percentage of married respondents revealed attempts to utilise other healthcare alternatives than their single, widowed, and divorced counterparts.

**Table 3:** The factors associated with attempting alternative services before visiting the ED.

Factor		Alternative services attempted before the ED visit		*p*-value
		Yes, *n* (%)	No, *n* (%)	
Predisposing characteristic				
Age	18–34	91 (43.3)	119 (56.7)	0.368
	35–49	62 (49.6)	63 (50.4)	
	50–64	22 (56.4)	17 (43.6)	
	65 and above	4 (57.1)	3 (42.9)	
Gender	Male	93 (46.5)	107 (53.5)	0.843
	Female	86 (47.5)	95 (52.5)	
Race	Malay	117 (43.3)	153 (56.7)	0.070
	Indian	45 (57.7)	33 (42.3)	
	Chinese	9 (42.9)	12 (57.1)	
	Others	8 (66.7)	4 (33.3)	
Marital status	Single	37 (35.6)	67 (64.4)	**0.003**
	Married	140 (52.4)	127 (47.6)	
	Widower/Widow/Widowed	2 (20.0)	8 (80.0)	
Employment status	Housewife	23 (46.0)	27 (54.0)	0.328
	Private worker	93 (54.4)	112 (54.6)	
	Self-employed	25 (52.1)	23 (47.9)	
	Pensioner	3 (42.9)	4 (57.1)	
	Public servant	25 (56.8)	19 (43.2)	
	Student	4 (23.5)	13 (76.5)	
	Unemployed	6 (60.0)	4 (40.0)	
Educational level	No formal education	4 (57.1)	3 (42.9)	0.945
	Primary school	9 (47.4)	10 (52.6)	
	Secondary school	102 (46.2)	119 (53.8)	
	Tertiary (College/University)	64 (47.8)	70 (52.2)	
Enabling characteristic				
Income	Under RM 4,850	153 (45.8)	181 (54.2)	0.207
	RM 4,851–RM 10,970	26 (56.5)	20 (43.5)	
	Over RM 10,970	0 (0.0)	1 (100.0)	
Insurance status	Yes	79 (59.8)	53 (40.2)	
	No	100 (40.2)	149 (59.8)	**<0.001**
Transportation to the ED	Own transport	165 (48.7)	174 (51.3)	0.092
	Public transport	6 (46.2)	7 (53.8)	
	Others	8 (27.6)	21 (72.4)	
Distance to ED	Mean (SD)	12.3 (777.8)	12.0 (839.0)	0.728
Time of visit	Office hour	107 (55.2)	87 (44.8)	**<0.001**
	After office hours	72 (38.5)	115 (61.5)	
Need factor				
Duration of illness onset	Under 24 h	51 (26.6)	141 (73.4)	**<0.001**
	1–3 days	81 (63.3)	47 (36.7)	
	4–7 days	29 (78.4)	8 (21.6)	
	1–4 weeks	18 (75.0)	6 (25.0)	
Perceived requirement for urgent treatment	Yes	140 (44.9)	172 (55.1)	0.079
	No	39 (56.5)	30 (43.5)	

*p*-values were calculated using the Chi-square test for independence. Bold values indicate statistical significance (*p* < 0.05).

According to the information gathered in this study, respondents seeking care after office hours were less likely to try alternative services. Furthermore, the duration of illness onset significantly influenced the decision, with shorter sickness periods resulting in fewer attempts to utilise alternative healthcare assistance. Insurance status also played a substantial role in the respondents’ visits to the ED. Respondents without insurance documented a higher percentage of not visiting healthcare facilities other than the ED than those with insurance. Nonetheless, other variables, such as age, gender, race, education, income, transportation to the ED, distance to the ED, and perceived requirement for urgent treatment, did not demonstrate statistically notable associations with attempting alternative healthcare services before ED presentation.

Table 4 lists the association between the respondents’ first time visit to the ED in the past 12 months with all parameters assessed in this study. Only two parameters demonstrated a significant association, marital (*p*-value = 0.001) and employment (*p*-value = 0.037) status. A higher percentage of married and widowed respondents attended the ED more frequently than single respondents. Housewives and unemployed respondents also visit the ED more frequently than the other groups.

**Table 4:** Factors associated with first-time visit to the ED in the past 12 months.

Factor		First time visit to the ED within 12 months		*p*-value
		Yes *n* (%)	No *n* (%)	
Predisposing characteristic				
Age	18–34	54 (25.7)	156 (74.3)	0.477
	35–49	26 (20.8)	99 (79.2)	
	50–64	6 (15.4)	33 (84.6)	
	65 and above	1 (14.3)	6 (85.7)	
Gender	Male	45 (22.5)	155 (77.5)	0.870
	Female	42 (23.2)	139 (76.8)	
Race	Malay	63 (23.3)	207 (76.7)	0.541
	Indian	15 (19.2)	63 (80.8)	
	Chinese	7 (33.3)	14 (66.7)	
	Others	2 (16.7)	10 (83.3)	
Marital status	Single	37 (35.6)	67 (64.4)	0.003
	Married	48 (18.0)	219 (82.0)	
	Widower/Widow/Widowed	2 (20.0)	8 (80.0)	
Employment status	Housewife	5 (10.0)	45 (90.0)	0.037
	Private worker	56 (27.3)	149 (72.7)	
	Self-employed	6 (12.5)	42 (87.5)	
	Pensioner	1 (14.3)	42 (85.7)	
	Public servant	13 (29.5)	31 (70.5)	
	Student	5 (29.4)	12 (70.6)	
	Unemployed	1 (10.0)	9 (90.0)	
Educational level	No formal education	1 (14.3)	6 (85.7)	0.131
	Primary school	1 (5.3)	18 (94.7)	
	Secondary school	48 (21.7)	173 (78.3)	
	Tertiary (College/University)	37 (27.6)	97 (72.4)	
Enabling characteristic				
Income	Under RM 4,850	77 (23.1)	257 (76.9)	0.980
	RM 4,851–RM 10,970	10 (27.1)	36 (78.3)	
	Over RM 10,970	0 (0.0)	1 (100.0)	
Insurance status	Yes	36 (27.3)	96 (72.7)	0.133
	No	51 (20.5)	198 (79.5)	
Transportation to the ED	Own transport	75 (22.1)	264 (77.9)	
	Public transport	2 (15.4)	11 (84.6)	
	Others	10 (34.5)	19 (65.5)	
Distance to ED	Mean (SD)	13.1 (894.0)	11.8 (782.4)	0.215
Time of visit	Office hour	46 (23.7)	148 (76.3)	0.678
	After office hours	41 (21.9)	146 (78.1)	
Need factor				
Duration of illness onset	Under 24 h	45 (23.4)	147 (76.6)	0.893
	1–3 days	30 (23.4)	98 (76.6)	
	4–7 days	8 (21.6)	29 (78.4)	
	1–4 weeks	4 (16.7)	20 (83.3)	
Perceived requirement for urgent treatment	Yes	72 (23.1)	240 (76.9)	0.811
	No	15 (21.7)	54 (78.3)	

### Factors refraining respondents from utilising alternative services before and frequently visiting the ED in the past 12 months with multiple logistic regression

Tables 5, 6 demonstrate the associations and likelihood of visiting alternative healthcare services before visiting the ED and the frequency of ED visits within 12 months. This study employed multiple logistic regression to assess the effects of confounding factors. The parameters selected for the analysis were based on *p*-values under 0.25 obtained from the univariable analysis.

**Table 5:** The multiple logistic regression results of the conditions contributing to respondents avoiding alternative healthcare services before visiting the ED.

Alternative services attempted before ED visit	*p*-value	Exp (B)	95% C.I. for EXP (B)	
			Lower	Upper
Race				
Others	Ref.			
Malay	0.301	2.24	0.49	10.34
Indian	0.519	1.68	0.35	8.15
Chinese	0.130	3.91	0.67	22.77
Marital status				
Single	Ref.			
Married	0.038	0.56	0.33	0.97
Widower/Widow/Widowed	0.772	1.31	0.21	8.14
Income				
Under RM 4,850	Ref.			
RM 4,851–10,970	>0.980	0.00	0.00	–
Over RM 10,970	>0.980	0.00	0.00	–
Insurance status				
Yes	0.004	0.47	0.28	0.79
Transportation to the ED				
Others	Ref.			
Own transport	0.189	0.52	0.20	1.38
Public transport	0.442	0.55	0.12	2.54
Time of visit				
Office hours	0.141	0.70	0.43	1.13
Duration of illness onset				
1–4 weeks	Ref.			
Less than 24 h	<0.001	9.06	3.17	25.87
1–3 days	0.120	2.34	0.80	6.82
4–7 days	0.774	1.21	0.33	4.39
Perceived requirement for urgent treatment				
Yes	0.220	1.46	0.80	2.67

**Table 6:** The multiple logistic regression results of the factors driving frequent visits to the ED within 12 months.

Multiple visits to the ED within 12 months	*p*-value	Exp (B)	95% C.I. for EXP (B)	
			Lower	Upper
Marital status				
Single	0.009			
Married	0.002	2.40	1.37	4.22
Widower/Widow/Widowed	0.330	2.26	0.44	11.67
Employment status				
Unemployed	0.242			
Housewife	0.793	0.73	0.07	7.76
Private worker	0.281	0.30	0.03	2.66
Self-employed	0.822	0.77	0.08	7.85
Pensioner	0.748	0.61	0.03	12.84
Public servant	0.275	0.28	0.03	2.74
Student	0.527	0.46	0.04	5.12
Educational level				
Tertiary (College/University)	0.560			
No formal education	0.530	2.04	0.22	18.83
Primary school	0.197	4.02	0.49	33.30
Secondary school	0.947	1.02	0.58	1.80
Insurance status				
Yes	0.375	0.78	0.45	1.35
Distance to the ED	0.250	0.98	0.95	1.01

All odds ratios reported are adjusted for relevant confounding variables included in the regression models.

The results of the multiple logistic regression showed that marital status, insurance status, and duration of illness significantly influenced respondents’ likelihood of not utilising alternative healthcare services before visiting the ED (*p* < 0.05). Married respondents had lower odds of not visiting alternative services compared to single respondents (AOR = 0.56), indicating a 44% higher likelihood of having sought alternative care prior to attending the ED. Possession of insurance was associated with 53% lower odds of seeking alternative care before the ED visit (AOR = 0.47). Respondents presenting with illness symptoms for less than 24 h were substantially more likely to visit the ED compared to those who had observed their illness for 1–4 weeks (AOR = 9.06, *p* < 0.001).

Regarding frequent ED visits within the past 12 months, after adjusting for confounding variables, only marital status remained a significant predictor. Married respondents had 2.4 times higher odds of frequent ED utilisation compared to single respondents (AOR = 2.40, *p* = 0.002). Some subgroups, such as widowers/widows and smaller employment or education categories, had relatively small sample sizes, which contributed to wide confidence intervals for certain odds ratios. These estimates should therefore be interpreted with caution, as they may reflect trends rather than precise effect sizes.

## Discussion

### Demographic and ED utilisation

This study was conducted at a public hospital in Malaysia. A total of 381 visitors to the ED were involved, contributing to a comprehensive demographic and healthcare utilisation profile. The respondents were predominantly between 18 and 34 years old, with a balanced gender distribution observed. Regarding ethnic representation, majority were Malays, which aligns with the nation’s general population demographics.

The respondents in this study were from varied socio-economic backgrounds. Most respondents were married and private sector employers. A notable prevalence in the B40 income group was also recorded, indicative of lower-income levels. The substantial number of respondents from the B40 income background seeking treatment at the ED highlighted significant socio-economic disparities in healthcare utilisation in Malaysia. The trend is also supported by global literature. Several reports consistently demonstrated that individuals with lower socio-economic status rely more on ED services (23). Patients from lower-income categories also commonly employ ED services as their primary care source (24).

Based on the findings of this study, majority of the respondents use personal transport, underscoring the importance of individual mobility. Conversely, a minimal number of respondents indicated reliance on public transport. Regarding patient arrival times at the ED, patterns in session 2 (2:00 p.m. to 7:00 p.m.) were observed as the peak visiting period. A significant number of respondents also came to the ED during session 3 (7:00 p.m. to 12:00 a.m.). The data aligned with the findings reported in a previous study (25). The arrival patterns reflected patient behaviour and provided insights for developing interventions to mitigate overcrowding and treatment delays. Similarly, most respondents seeking medical attention within the first 24 h supported the findings in another report (26), emphasising the perceived urgency of healthcare requirements. The prevalence of fever, cough, and cold reported as health concerns by the respondents also provided insights into common health issues encountered in the population (27).

This study assessed the motivations driving the respondents to seek treatment in the ED of a public hospital. Amongst the predominant factors influencing patient decisions are perceived severity and immediate health assistance requirements, which aligns with existing literature (28). Furthermore, trust in the accuracy of health assessments and confidence in the quality of care provided by the ED underscored the significance of perceived healthcare competence during emergencies (28). The respondents also revealed that proximity and affordability considerations resonated with the broader discourse on accessibility and cost as crucial determinants of healthcare utilisation (29). Meanwhile, a notable proportion indicated that visiting the ED was a standard practise. The data supported previous studies that highlighted the role of social norms in shaping healthcare-seeking behaviour (30).

### Factors influencing ED services utilisation

This study employed Andersen’s behavioural model of healthcare utilisation to examine the factors influencing visits to the emergency department (ED). The model enabled the identification of predisposing, enabling, and need factors shaping utilisation patterns. Behavioural insights were not part of the study design or analysis; rather, they are introduced in the discussion to provide a complementary lens for interpreting healthcare-seeking behaviours. Explanations drawing on concepts such as cognitive biases, heuristics, and social norms are therefore theoretical in nature and serve to contextualise the observed associations, rather than representing direct measurements from the data.

The outcomes highlighted marital status as a critical predisposing factor contributing to healthcare decisions, particularly in selecting ED services employment (30). From a behavioural insights perspective, social influences and social networks are central in shaping individuals’ healthcare-seeking behaviours (6). Married individuals are often embedded within strong social support structures, which can shape their healthcare-seeking behaviours. Such networks may encourage the use of alternative services before attending the ED, as advice and reassurance from family or peers can help manage non-urgent concerns (6). In this study, married respondents were more likely to use the ED than their single counterparts, despite potential support. This may reflect other factors associated with marital status, such as differences in health needs, care-seeking behaviour, or household responsibilities, rather than a higher burden of chronic illness per se (31). These contextual factors may partly account for the observed pattern. Within Andersen’s behavioural model, marital status acts as a predisposing factor that interacts with need-based drivers such as chronic illness and perceived urgency. Although enabling resources like family support could reduce ED reliance, the findings suggest that need factors may override these supports, underscoring the complex interplay of predisposing, enabling, and need factors in shaping ED use.

Decision-making tendencies highlighted by behavioural insights provide a complementary perspective on ED utilisation. The status quo bias may lead individuals to perceive the ED as the default and most dependable option during a health episode, particularly when access to primary care is perceived as difficult. Similarly, the availability bias may influence choices, as previous experiences of prompt treatment in the ED can reinforce the perception of the ED as the most convenient and accessible service (32, 33). These findings suggest that demographic factors, structural circumstances, and behavioural tendencies together contribute to the higher ED utilisation observed amongst married individuals. Whilst the associations identified in this study are not causal, they underscore the need for further research to disentangle the relative contributions of social, clinical, and behavioural mechanisms, which could inform targeted interventions to optimise ED use.

The perceived necessity for urgent care, particularly when involving children, can lead to an urgency bias and repeated ED visits, specifically amongst married parents. An overestimation of urgency may arise from the emotional weight of caring for a child and the fear of not acting quickly enough (loss aversion) (33), resulting in married individuals demonstrating an increased likelihood to make repeated ED visits. The marital responsibility felt by married parents can further compound and contribute to a heightened sense of urgency when a child’s health is involved. Previous studies also recognised the intricate interplay between social relationships and healthcare decisions, highlighting the necessity for tailored interventions that consider these relational dynamics (34). Recognising these patterns can inform strategies such as targeted education, decision-support tools, and communication campaigns to help parents accurately assess urgency and reduce non-essential ED visits.

According to the study’s findings, insurance status significantly influenced healthcare-seeking decisions, consistent with existing literature. Insured respondents were more likely to seek alternative healthcare services before visiting the ED, supporting evidence that insurance coverage facilitates access to primary care services (35). From a behavioural insights perspective, insured individuals face lower friction costs when accessing healthcare alternatives compared with those without insurance. This enables them to utilise alternative services without substantial out-of-pocket concerns, reducing the mental effort involved in decision-making. These observations align with nudge theory, which posits that individuals encountering fewer perceived barriers such as cost, access, or effort are more likely to choose options that avoid the ED (36). Furthermore, insured individuals may have greater confidence in accessing timely, appropriate care, which diminishes the perceived urgency of ED visits (37).

Uninsured individuals may fear unaffordable healthcare, leading them to view the ED as the only viable option for urgent care (35, 37). This behaviour aligns with the availability heuristic, whereby individuals rely on the most familiar and accessible option, in this case, the ED (26). Within Andersen’s behavioural model, lack of insurance represents a limitation in enabling resources, which constrains access to alternative care options. Concerns about high medical costs can therefore delay or discourage the use of other services perceived as inaccessible or unaffordable (38). Consequently, uninsured patients often turn to the ED for convenience and because enabling resources fail to support other avenues of care (39). Nevertheless, the insurance-related findings presented here should be interpreted with caution. This study was conducted in a single public hospital and did not include private hospitals, where many insured patients in Malaysia typically seek care due to advantages such as comfort, faster services, and ease of claims. As a result, the patterns observed in this sample may not fully reflect system-wide insurance utilisation behaviours. Future multi-setting studies are needed to determine whether these findings can be generalised to other contexts and patient populations.

According to responses obtained in this study, the duration of illness onset was a significant factor influencing respondents’ decisions to refrain from seeking alternative services before presenting to the ED. This finding aligns with previous reports emphasising that the perceived urgency of a condition drives patients to choose the ED, even when more appropriate alternatives exist (40). One study noted that whilst many patients with less serious medical concerns consider alternative care options, most still perceive the ED as the optimal setting for care (41), with uncertainty about primary care alternatives cited as a key reason (42). Within Andersen’s behavioural model, this reflects the role of need factors, specifically perceived urgency, which can override enabling resources such as the availability of primary care. When illness symptoms are prolonged or escalating, patients may interpret the situation as requiring immediate attention, explaining their decision to bypass alternative services and present directly to the ED.

From a behavioural insights perspective, decisions to visit the ED are influenced by several psychological and social factors, including cognitive biases and heuristics (43). According to the availability heuristic, the ED is the most familiar and easily accessible option for individuals seeking urgent health assistance. This reliance on readily available options can lead patients to overlook alternative services, even when these might be more appropriate, because the ED is perceived as the “default” solution (44, 45). Understanding this behavioural tendency highlights the potential for interventions, such as targeted nudges, educational campaigns, and decision-support tools, to guide patients towards more suitable care options and reduce unnecessary ED utilisation.

The perception of severity strongly influences healthcare-seeking decisions. Several patients in this study reported not seeking alternative care because they believed their condition was too serious to be managed outside the ED. This behaviour can be understood through the lenses of risk perception and loss aversion. According to these theories, patients choose the ED as the “safe” option, fearing the potential consequences of delaying care or misjudging the severity of their condition. This common behavioural bias leads individuals to err on the side of caution, even when the probability of requiring emergency care is low (46). Within Andersen’s behavioural model, this represents a need factor perceived urgency that can override enabling resources, such as primary care availability, driving patients to default to emergency services even for non-urgent concerns. Recognising this bias highlights opportunities for targeted interventions, such as education and decision-support tools, to recalibrate perceptions of urgency and guide more appropriate use of healthcare services.

Limited access to primary care and inadequate awareness of alternative care pathways contribute to individuals opting to visit the ED. Patients may not be aware of other available options, such as urgent care centres or telemedicine services, which could provide more timely and appropriate care for their health concerns (46), potentially leading to a default reliance on the ED. Integrating the findings from this study into behavioural insights-based interventions, such as nudging, offers a practical approach to improving efficiency, responsiveness, and public engagement in healthcare service delivery (47, 48). Previous studies have demonstrated that behavioural strategies can increase clinical workflow and patient throughput whilst reducing no-shows in EDs (49, 50). Consequently, the insights from this study can be applied to optimise emergency response efficiency, guide resource allocation, and inform targeted interventions to reduce non-urgent ED visits.

Beyond interpreting the findings, this study offers several practical and policy implications to guide strategies for addressing non-urgent ED utilisation. The temporal distribution of visits underscores the need to align staffing and resources with peak visiting times. Streamlining fast-track pathways for patients triaged to the green zone may help minimise congestion whilst ensuring that urgent cases receive timely care (51). Since insured patients may prefer private healthcare settings, clearer signposting to appropriate primary or urgent care services is recommended. Strengthening referral pathways and providing transparent information about alternative service availability could reduce unnecessary ED attendance (52). These recommendations are informed directly by the study findings, highlighting how behavioural, structural, and accessibility factors can be targeted to optimise ED use and improve healthcare system efficiency.

The findings from this study carry several important implications for policy and practise. From a service organisation perspective, aligning staffing levels and resources with peak visiting times could help mitigate delays and overcrowding. Hospitals may also consider streamlining fast-track pathways for green zone patients to improve efficiency whilst ensuring that urgent cases remain prioritised. Another critical implication relates to care navigation. Many patients in this study reported convenience and accessibility as key drivers of ED use, highlighting the need to translate these findings into targeted interventions. Strengthening primary care alternatives and enhancing referral systems are essential. Clearer signposting to appropriate primary or urgent care services, particularly for insured patients who may prefer private settings, could reduce unnecessary reliance on public EDs. Expanding primary care capacity more broadly, including after-hours access, may also divert non-urgent cases from emergency departments.

Equally important is communication. Misperceptions about the urgency of health issues continue to be a common driver of ED attendance. Targeted health communication strategies can recalibrate perceptions of urgency and reshape social norms that normalise ED visits for non-urgent concerns. Evidence suggests that well-designed community-based interventions can reduce emergency admissions and improve patient outcomes (53). Finally, improving data integration and feedback loops between EDs and primary care providers could help reduce repeat non-urgent visits. Incorporating ED visit summaries into primary care records would strengthen continuity of care, facilitate timely follow-up, and reduce dependence on emergency services for conditions that are more appropriately managed elsewhere (54). This findings provide actionable insights for policymakers and healthcare providers by highlighting specific factors such as trust in care, proximity, and behavioural drivers that can be targeted to optimise interventions. Taken together, these recommendations highlight the need for a multi-pronged approach that combines service organisation, navigation support, communication, and data integration. Such measures could help health systems manage non-urgent ED use more effectively, ease pressure on emergency services, and ultimately improve both efficiency and patient experiences.

Several limitations must be acknowledged. The study was conducted in a single public hospital and focused exclusively on patients in the green zone, limiting its generalizability to other triage categories, hospitals, and the wider healthcare system. The study was conducted amongst individuals triaged to the green zone who were willing to participate and complete the questionnaire. It is acknowledged that including only participants who consented may introduce selection bias, as these individuals might differ systematically from those who declined participation. In addition, the study focused on a single public hospital, which may limit generalisability. However, despite these limitations, the findings provide a meaningful and valuable foundation for understanding patterns of ED utilisation, offering insights that can inform future research and policy interventions in similar settings. Future multi-site studies incorporating wider triage categories and additional determinants will further strengthen this evidence base. Furthermore, potential confounders such as health literacy, socioeconomic status, and delayed presentation could have introduced bias and influenced the observed associations. Whilst behavioural insights were not directly measured in the study design, they were applied in the discussion to interpret healthcare-seeking behaviours. Concepts such as social norms, cognitive biases, and perceived urgency provide a complementary perspective that may help explain the observed associations. These interpretations remain theoretical but can generate new hypotheses and inform the development of future interventions.

## Conclusion

This study provides valuable insights into factors influencing emergency department (ED) utilisation in Malaysia, guided by Andersen’s behavioural model of healthcare utilisation. Amongst 381 respondents, predisposing (marital status), enabling (insurance coverage), and need factors (duration of illness) were significantly associated with ED use, whilst trust in care quality and proximity of the facility also emerged as key motivations. These findings underscore the complex interplay between individual characteristics, perceived healthcare needs, and structural determinants in shaping healthcare-seeking behaviour. Although the study focused on a single public hospital and green-zone patients, limiting generalisability, the results offer a foundation for understanding patterns of non-urgent ED use. Integrating behavioural insights, including social norms, cognitive biases, and perceived urgency, provides a complementary perspective for interpreting these behaviours and designing interventions. Future research should expand to multiple hospitals, diverse triage categories, and additional confounding factors to strengthen evidence for comprehensive strategies. Practically, these findings highlight actionable steps for policymakers and healthcare providers: enhancing primary care accessibility, improving public awareness of appropriate ED use, and implementing targeted interventions for populations at higher risk of frequent ED visits. By translating these insights into policy and practise, this study contributes to promoting appropriate healthcare-seeking behaviour, optimising ED efficiency, and improving the overall performance of Malaysia’s public healthcare system.

## Data Availability

The raw data supporting the conclusions of this article will be made available by the authors, without undue reservation.
